# Golgi-Localized OsFPN1 is Involved in Co and Ni Transport and Their Detoxification in Rice

**DOI:** 10.1186/s12284-022-00583-3

**Published:** 2022-07-11

**Authors:** Manman Kan, Toru Fujiwara, Takehiro Kamiya

**Affiliations:** 1 Department of Applied Biological Chemistry, Graduate School of Agricultural and Life Sciences, The University of Tokyo, 1-1-1 Yayoi, Bunkyo-ku, Tokyo, 113-8657 Japan; 2 Precursory Research for Embryonic Science and Technology, Japan Science and Technology Agency (JST), 4-1-8 Honcho Kawaguchi, Saitama, 332-0012 Japan

**Keywords:** Cobalt, Detoxification, Ferroportin (FPN), Nickel, Rice, Root to shoot

## Abstract

Cobalt (Co) and nickel (Ni) are beneficial and essential elements for plants, respectively, with the latter required for urease activity, which hydrolyzes urea into ammonium in plants. However, excess Co and Ni are toxic to plants and their transport mechanisms in rice are unclear. Here, we analyzed an ethyl methanesulfonate (EMS)-mutagenized rice mutant, *1187_n*, with increased Co and Ni contents in its brown rice and shoots. *1187_n* has a mutation in *OsFPN1*, which was correlated with a high Co and Ni phenotype in F_2_ crosses between the parental line and mutant. In addition, *CRISPR/Cas9* mutants exhibited a phenotype similar to that of *1187_n*, demonstrating that *OsFPN1* is the causal gene of the mutant. In addition to the high Co and Ni in brown rice and shoots, the mutant also exhibited high Co and Ni concentrations in the xylem sap, but low concentrations in the roots, suggesting that *OsFPN1* is involved in the root-to-shoot translocation of Co and Ni. The growth of *1187_n* and *CRISPR/Cas9* lines were suppressed under high Co and Ni condition, indicating *OsFPN1* is required for the normal growth under high Co and Ni. An *OsFPN1*-green fluorescent protein (GFP) fusion protein was localized to the Golgi apparatus. Yeast carrying *GFP-OsFPN1* increased sensitivity to high Co contents and decreased Co and Ni accumulation. These results suggest that OsFPN1 can transport Co and Ni and is vital detoxification in rice.

**Supplementary Information:**

The online version contains supplementary material available at 10.1186/s12284-022-00583-3.

## Background

Co and Ni are rare elements of the earth’s crust and usually compete with Fe when absorbed by organisms (Buschow 1971; Salnikow et al. 2004; Morrissey et al. 2009). Co is a beneficial element that improves growth in certain plants, particularly leguminous plants (Karlengen et al. 2013). However, excess Co leads to black patches in tomato fruit (Chatterjee and Chatterjee 2005), and chlorosis in the young leaves of French bean (*Phaseolus vulgaris L.*), groundnut mung bean (*Vigna radiate*), and tomato (*Lycopersicon esculentum*) plants by causing reduced chlorophyll a, b, and carotene concentrations (Liu et al. 2000; Gopal et al. 2003; Chatterjee et al. 2006). Ni is an essential micronutrient in plants that is present in the active site of urease, an enzyme that hydrolyzes urea into ammonium in plant tissues (Eskew et al. 1983, 1984; Kerby et al. 1997). In addition to urease, Ni forms a part of other metalloenzymes, such as superoxide dismutase, glyoxalase, and hydrogenase. Similar to Co, excess Ni affects plant germination and growth. For example, the application of 1.5 mM of Ni decreased pigeon pea (*Cajanus cajan* (L.) *Millspaugh*) germination by approximately 20% (Aggarwal et al. 1990; Rao and Sresty 2000). Additionally, the growth of cabbage (*Brassica oleracea* L var. capitate) was inhibited by 0.5 mM of Ni (Pandey and Sharma 2002).

Ferroportin (FPN), also referred to as iron-regulated gene1 (IREG1) or metal transporter protein1, was first identified in hypotransferrinemic mice, where it was involved in Fe absorption in the duodenum (McKie et al. 2000) and Fe recycling in macrophages (Muckenthaler et al. 2008). *Arabidopsis thaliana* contains three homologous genes of mammalian *FPN*, i.e., *AtFPN1*, *AtFPN2*, and *AtFPN3*. AtFPN1 encodes a plasma membrane-localized exporter of Co and Fe, which is expressed in the vasculature of the root and shoot and is involved in the loading of these elements from the pericycle to the xylem. (Morrissey et al. 2009). The expression of *AtFPN1* is not regulated by Fe (Morrissey et al. 2009), while *AtFPN2 and AtFPN3* is induced by Fe starvation (Morrissey et al. 2009; Kim et al. 2021). *AtFPN2* is localized in the tonoplast and is expressed in the epidermis and cortex of the root (Morrissey et al. 2009). The *AtFPN2* mutant causes high Co and Ni accumulation in the shoots, but low accumulation in the roots, indicating that it sequesters Co and Ni into vacuoles in the root for detoxification (Schaaf et al. 2006; Morrissey et al. 2009). AtFPN3, which has 20% similarity to AtFPN1 and AtFPN2, is localized in chloroplasts/mitochondria function as Fe exporter and essential for Fe homeostasis (Kim et al. 2021). In addition to Fe homeostasis, AtFPN3, also known as MAR1, also played a role in controlling the entry of antibiotics into chloroplasts (Conte et al. 2010; Conte and Lloyd 2010). In the hyperaccumulator *Psychotria gabriellae*, the expression of *PgFPN1* in mRNA expression was higher than that in non-accumulator species (Merlot et al. 2014). The overexpression of *PgFPN1* in Arabidopsis enhances its tolerance to high levels of Ni (Merlot et al. 2014). In monocotyledons, buckwheat (*Fagopyrum esculentum Moench*) *FPN1* (*FeFPN1*) is involved in internal Al^3+^ detoxification (Yokosho et al. 2016). *FeFPN1* is expressed in the roots and is greatly upregulated by Al^3+^, but not by Fe, unlike *AtFPN2* (Schaaf et al. 2006; Morrissey et al. 2009). The overexpression of *FeFPN1* in Arabidopsis confers Al tolerance, suggesting that it is involved in Al detoxification in buckwheat (Yokosho et al. 2016). However, the function of putative FPNs in rice remains unclear.

In this study, we conducted ionome screening of an ethyl methanesulfonate (EMS)-mutagenized rice M_2_ population and identified a mutant with high Co and Ni contents in the grain and shoot. The causal gene encoding FPN is *OsFPN1*, which is localized to the Golgi apparatus and mediates Co and Ni transport. The mutants exhibited sensitivity to high levels of Co and Ni, indicating that OsFPN1 is necessary for tolerance to these elements.

## Results

### *1187_n* Exhibited Increased Co and Ni Contents in Brown Rice, Shoot, and Xylem Sap

**Fig. 1:**
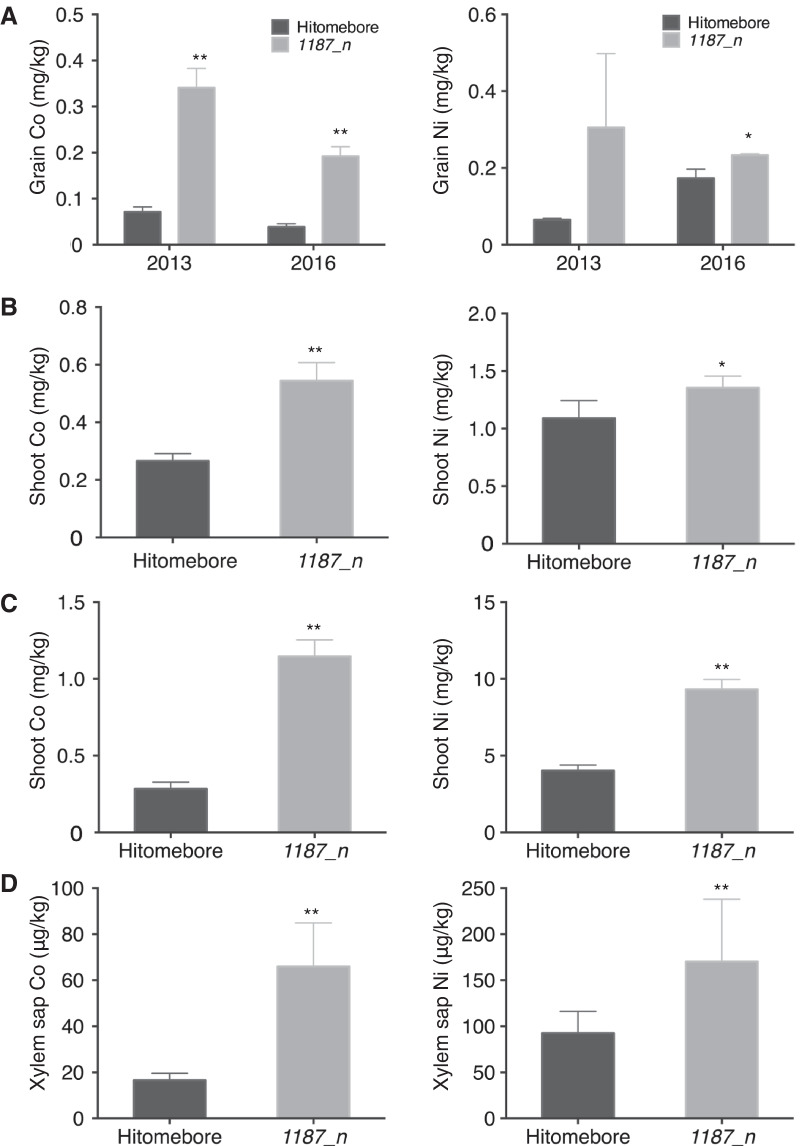
Co and Ni accumulation in Hitomebore and *1187_n*. **A** Co and Ni concentrations in the grains of Hitomebore and *1187_n* grown in a paddy field (n = 3–4). **B** Co and Ni concentrations in the shoots of Hitomebore and *1187_n* cultivated in soil (n = 3–4). **C** Co and Ni concentrations in the shoots of Hitomebore and *1187_n* cultivated in a hydroponic culture (n = 11–12). **D** Co and Ni concentrations in the xylem sap of Hitomebore and *1187_n* cultivated in a hydroponic culture (n = 9–11). Xylem sap was collected for 4 h. For **A**, **B** data are the same as those in Figure S1. For **B**–**D** 21-d-old seedlings were harvested for the experiments. Data represents the mean ± SD. Student’s *t*-test, *p < 0.05; **p < 0.01

To identify the genes regulating mineral transport in rice (*O. sativa* cv. Hitomebore), ionome screening of the EMS-mutagenized M_2_ population was conducted (Tanaka et al. 2016). We isolated a mutant, *1187_n*, which exhibited high Co and Ni concentrations in brown rice grown in paddy fields during 2013 and 2016 (Additional file 1: Fig. S1, Fig. 1A). Additionally, *1187_n* exhibited high Co and Ni concentrations in shoots when the plants were grown in soil (Fig. 1B) or the Kimura B hydroponic solution (Ma et al. 2001) (Fig. 1C). Additionally, increased Co and Ni concentrations were observed in the xylem sap (Kan et al. 2019) of *1187_n* grown under hydroponic conditions (Fig. 1D). These results indicated that *1187_n* has defect in root-to-shoot transport of Co and Ni.

### Sensitivity of *1187_n* to High Co and Ni

**Fig. 2:**
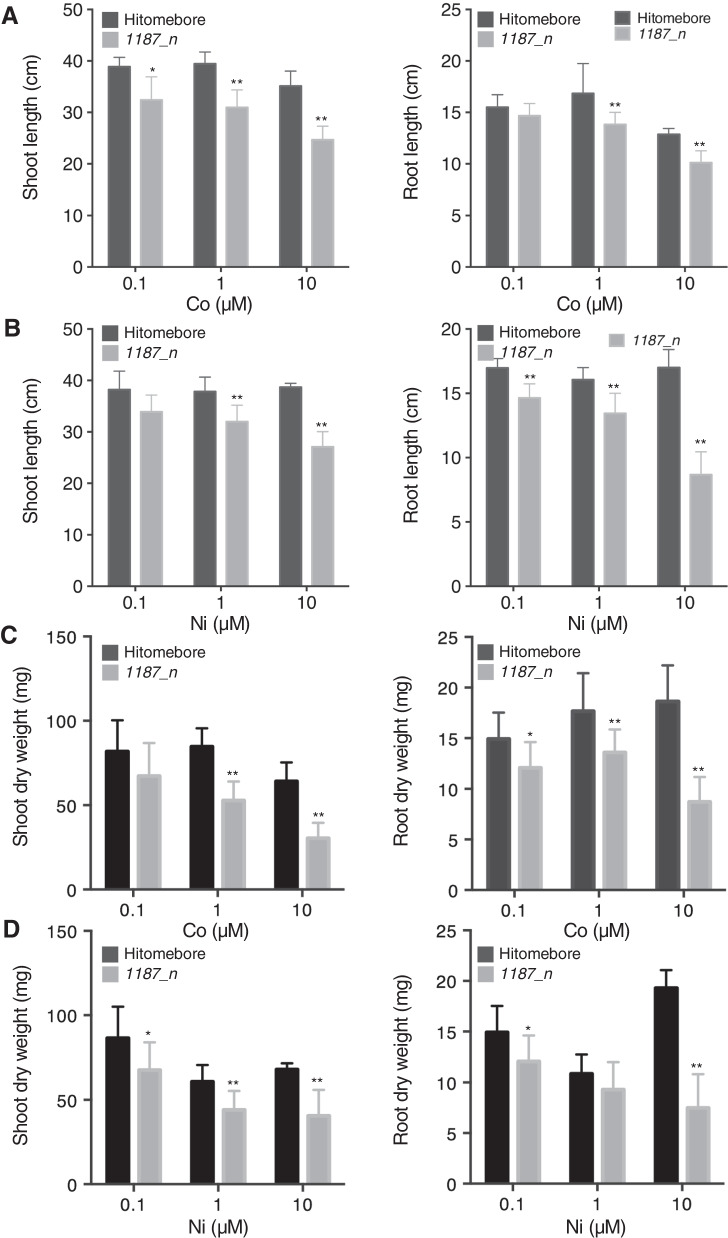
Growth phenotype of Hitomebore and *1187_n* under various Co and Ni conditions. Shoot (left panel) and root (right panel) lengths (**A**, **B**) and dry weight (**C**, **D**) under various Co (**A**) and Ni (**B**) conditions. Plants were grown in hydroponic culture for three weeks. Data represent the means ± SD (n = 8–11). Student’s *t*-test, *p < 0.05; **p < 0.01

To observe the effects of Co and Ni on the growth of *1187_n*, the plants were grown in Kimura B solution supplemented with various concentrations of Co or Ni for three weeks. The seedlings were cut at the shoot–root junction to divide them into shoots and roots, and the length and dry weight of the shoots and roots were measured. The shoot and root growth of *1187_n* were more suppressed under applied Co or Ni concentrations of 1 and 10 μM compared to the wild type (Fig. 2). These results indicate that *1187_n* is sensitive to high levels of Co and Ni.

### *OsFPN1 *is the Causal Gene of *1187_n*

**Fig. 3:**
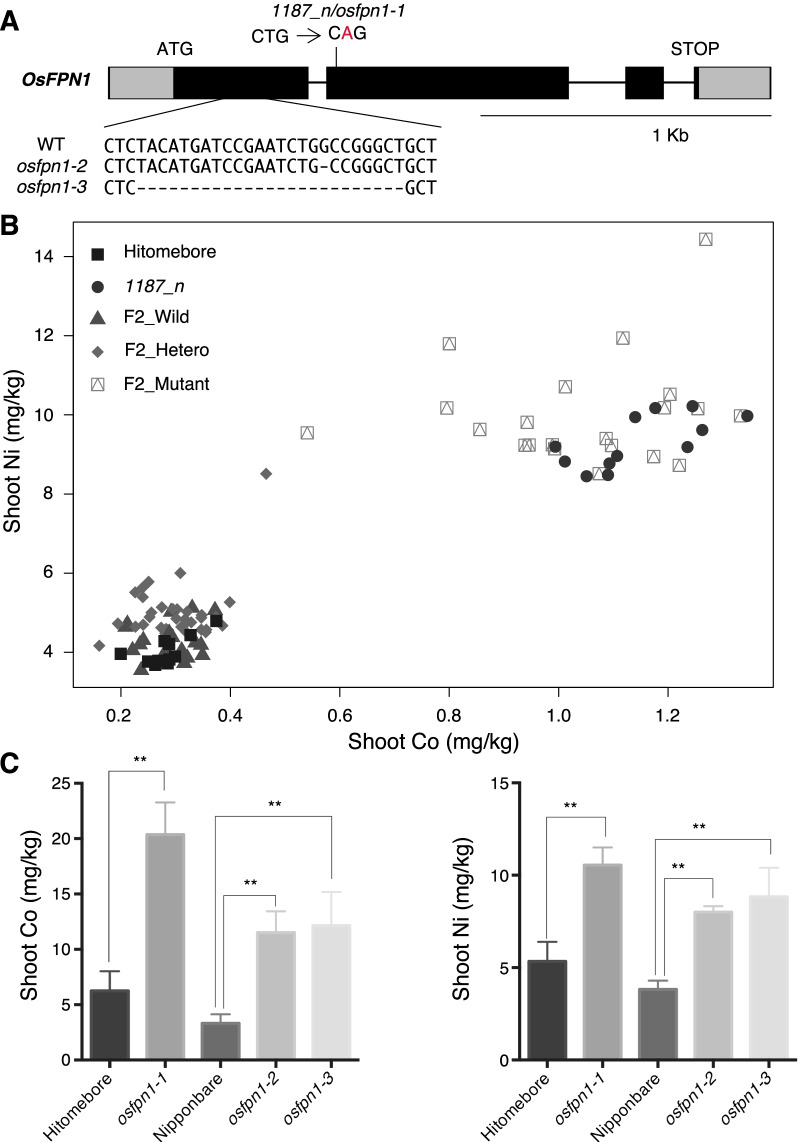
Responsibility of *OsFPN1* for the phenotype of *1187_n.* **A** Exon intron structure of *OsFPN1*. Gray boxes, dark boxes, and lines represent the untranslated region, exon, and intron, respectively. *OsFPN1* of *1187_n* has a nonsynonymous mutation of T to G [leucine (L) is replaced with glutamine (G)]. *Osfpn1-2* and *osfpn1-3* are CRISPR/Cas 9 mutants with 1 bp and 26 bp deletions at the first exon of *OsFPN1*, respectively. WT (Nipponbare) is the background of the mutants. **B** Scatterplot based on the Co and Ni concentrations in the shoots of three-week-old seedlings of Hitomebore, *1187_n*, and F2. F2_Wild, F2_Hetero, and F2_Mutant represent the wild-type, heterozygous, and mutant homozygous genotypes of F2, respectively. **C** Co and Ni concentrations in the shoots of Hitomebore, *osfpn1-1*, Nipponbare, *osfpn1-2,* and *osfpn1-3*. Plants were grown in Kimura B solution supplied with 10 trace elements. Data represent the means ± SD (n = 3–12). Student’s *t*-test (compared to Hitomebore or Nipponbare). **p < 0.01

The phenotype of increased Co concentration in the shoot and sensitivity to Co and Ni of *1187_n* is similar to the phenotype of the *fpn2* mutant of *A. thaliana* (Morrissey et al. 2009). Thus, we hypothesized that the FPN2 homolog in rice was the causal gene of *1187_n*. Rice contains only one homolog, *Os06g0560000* (hereafter named *OsFPN1*) (Additional file 1: Fig. S2). Sequencing analysis of *OsFPN1* in *1187_n* revealed that *1187_n* had a mutation (T to G) in the second exon, which caused Leu^181^ to Gln substitution (Fig. 3A). The OsFPN1 protein was predicted to be composed of nine transmembrane domains (Additional file 1: Fig. S3A). Leu^181^ was located between transmembrane domains 4 and 5, and was highly conserved among plant and human FPN homologs (Additional file 1: Fig. S3).

To confirm whether *OsFPN1* was the gene responsible for the increased Co and Ni in *1187_n*, ionome analysis of F_2_ crosses between *1187_n* and wild type was conducted in seedlings grown under hydroponic conditions (Fig. 3B). The Co and Ni concentrations were positively correlated and segregated with a ratio of 56:20 (wild type:mutant phenotype), which fits the 3:1 ratio (chi-squared test, *χ*^2^ = 0.853), indicating that increased levels of both Co and Ni were caused by a single recessive gene (Fig. 3B). Next, to observe the correlation between the ionome phenotype and mutation in *OsFPN1*, we designed a dCAPS marker to detect the mutation, and the F_2_ plants used for ICP-MS analysis underwent genotyping (Fig. 3B). All F_2_ seedlings with homozygous mutations exhibited increased Co and Ni contents, while the wild-type homozygous and heterozygous F_2_ seedlings exhibited low Ni and Co contents (Fig. 3B). These results indicate the co-segregation between the Co and Ni concentrations and the genotype, suggesting that *OsFPN1* is the causal gene of *1187_n*. Hereafter, we refer to*1187_n* as *osfpn1-1.*

To demonstrate that *OsFPN1* is the causal gene, two independent CRISPR/Cas9 lines, *osfpn1-2* and *osfpn1-3*, were generated in Nipponbare as it was difficult to transform Hitomebore. *osfpn1-2* and *osfpn1-3* have 1 bp and 26 bp deletions, respectively. First, we conducted ionome analysis in seedlings, and found that *osfpn1-1*, *osfpn1-2*, and *osfpn1-3* exhibited higher Co and Ni contents in their shoots than the wild-type plants (Fig. 3C). Taken together with the correlation between the phenotype and genotype (Fig. 3B), these data demonstrate that *OsFPN1* is the causal gene of *1187_n*.

According to the RiceXPro database (https://ricexpro.dna.affrc.go.jp/) (Sato et al. 2011), *OsFPN1* is expressed in whole tissues, especially high in root and reproductive organs.

### *Osfpn1* Mutants Were Sensitive to High Co and Ni

**Fig. 4:**
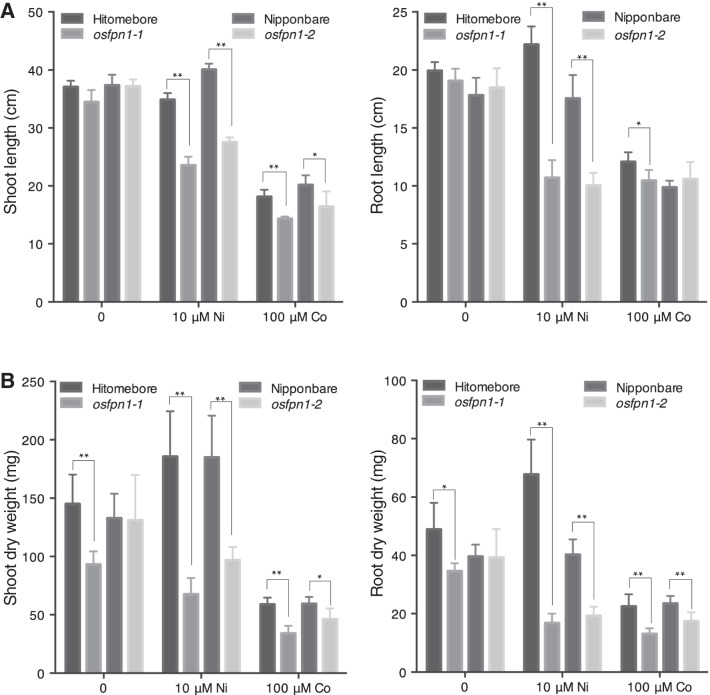
Sensitivity of *osfpn1-1* and *osfpn1-2* to high Co and Ni. Shoot (left panel) and root (right panel) lengths (**A**) and dry weight (**B**) of one-week-old plants grown in Kimura B solution transferred without Co or Ni application, or 10 or 100-µM Ni application in Kimura B solution for two weeks. Data represent the means ± SD (n = 5). Student’s *t*-test (compared to Hitomebore or Nipponbare). **p < 0.01, *p < 0.05

To investigate whether the sensitivity of *osfpn1-1 (1187_n*) is also caused by the mutation in *OsFPN1*, we tested the tolerance of *osfpn1-1* and *osfpn1-2,* together with their wild type, to Co and Ni (Fig. 4). Without Co or Ni, the shoot and root growth of *osfpn1-1* and *osfpn1-2* were similar to that of the wild type. Under 10 μM Ni, the shoot and root growth of *osfpn1-1* and *osfpn1-2* were inhibited. These results indicated that *OsFPN1* is important for high Co and Ni tolerance.

### *OsFPN1* is Localized to the Golgi Apparatus

**Fig. 5:**
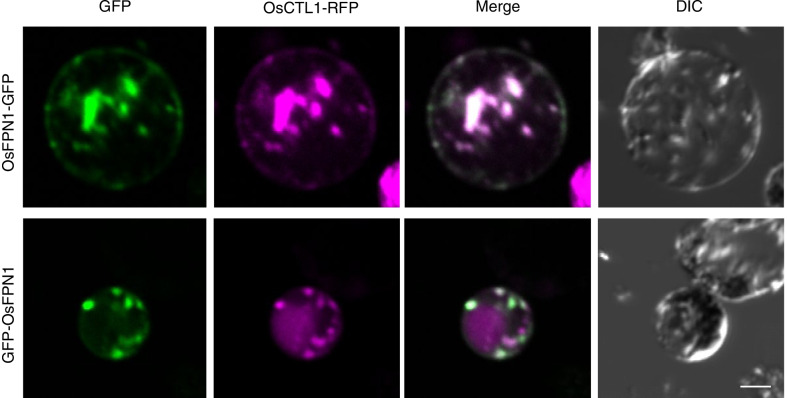
Subcellular localization of OsFPN1. Transient expression of *OsFPN1-GFP* and *GFP-OsFPN1* in protoplasts prepared from 10-d-old rice leaf sheaths. *OsCTL1-RFP* was used as the Golgi marker. Bar = 10 µm

*OsFPN1* is a membrane protein with nine putative transmembrane domains (Additional file 1: Fig. S2). To determine the subcellular localization of *OsFPN1*, *OsFPN1-GFP* or *GFP-OsFPN1* were driven by the CaMV 35S RNA promoter and transiently expressed in protoplasts prepared from rice leaf sheaths. In the protoplast expressing *OsFPN1*, a dot-like structure was observed in both the N- and C-terminal fusion proteins. The dot-like structure was co-localized with the Golgi marker *OsCTL1* (Wu et al. 2012) (Fig. 5). Therefore, *OsFPN1-GFP* was localized to the Golgi apparatus.

### OsFPN1 can Transport Co and Ni in Yeast

**Fig. 6:**
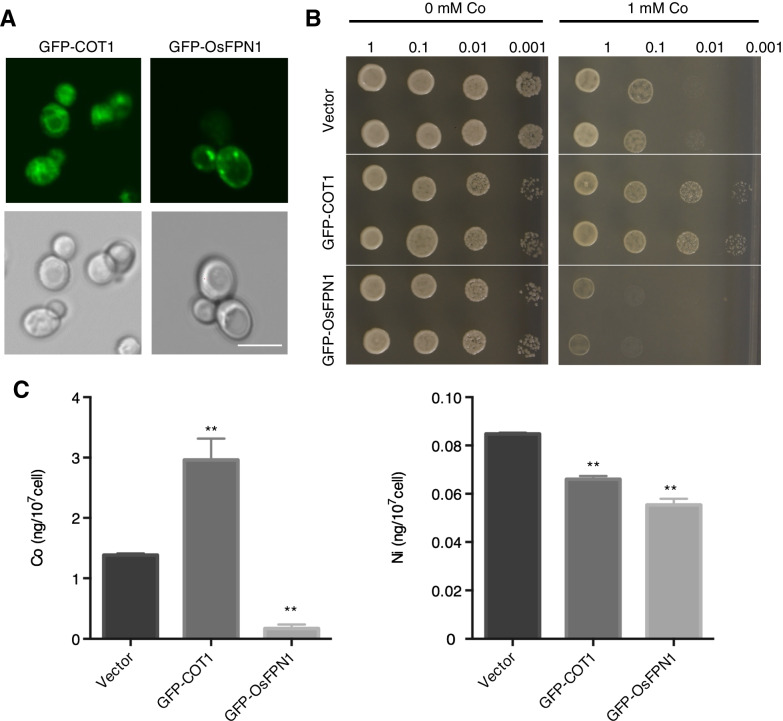
Functional characterization in yeast. **A** *GFP-COT1* and *GFP-OsFPN1* localization in the *cot1* yeast mutant. Bar = 10 µm. **B** High Co tolerance assay of yeast used in **A**. Yeast cells were adjusted to the indicated OD and spotted on SD-uracil medium containing 0 or 1 mM of Co. Images were captured after 2 d of incubation. **C** Co and Ni concentration in *cot1* expressing *GFP-COT1* and *GFP-OsFPN1*. Yeasts were incubated with 50 µM of Co or Ni for 1 h, and Co and Ni were determined by ICP-MS. n = 3–6. Dunnett’s test compared to the vector, **p < 0.01

To test whether *OsFPN1* can transport Co and Ni, *GFP-OsFPN1* was expressed in Co-and Ni-sensitive yeast *cot1* mutants (Schaaf et al. 2006; Morrissey et al. 2009). COT1p is a Zn and Co transporter localized in vacuoles, and *cot1* mutants are sensitive to high Co concentrations (Conklin et al. 1992). In the following experiments, *COT1* was used as a positive control and GFP-fused *COT1* (GFP-COT1) was introduced into the yeast. GFP fluorescence was observed in the yeast transfected with *GFP-COT1* and *GFP-OsFPN1*, suggesting that the genes were translated (Fig. 5A). Based on the pattern of GFP, GFP-COT1 could be localized to the vacuole, which is consistent with a previous report (Morrissey et al. 2009), and GFP-OsFPN1 was observed in the dot-like structure and cell periphery (Fig. 6A).

To determine the Co tolerance of *cot1* carrying each construct, the yeasts were spotted onto SD-uracil free media containing a high (1 mM) Co concentration. Consistent with a previous report (Morrissey et al. 2009), *COT1-GFP* conferred tolerance to 1 mM of Co (Fig. 6B). However, the expression of *OsFPN1-GFP* increased Co sensitivity when compared to the empty vector (Fig. 6B). We also determined the Co concentrations of yeast carrying *GFP-OsFPN1* after incubation in liquid media. The yeast carrying *GFP-OsFPN1* exhibited a lower Co concentration than that carrying the empty vector, while it was higher in the yeast carrying *GFP-COT1* (Fig. 6C). These results suggest that *OsFPN1* can transport Co in yeast. We also determined the Ni concentration in the yeasts after incubation with SD-uracil free media containing Ni, and the yeast carrying *GFP-OsFPN1* exhibited a lower Ni content than that carrying the empty vector (Fig. 6C), suggesting that OsFPN1 can transport both Ni and Co in yeast.

### *OsFPN1* is Involved in Co and Ni Translocation, Particularly Under Low-Fe Conditions

**Fig. 7:**
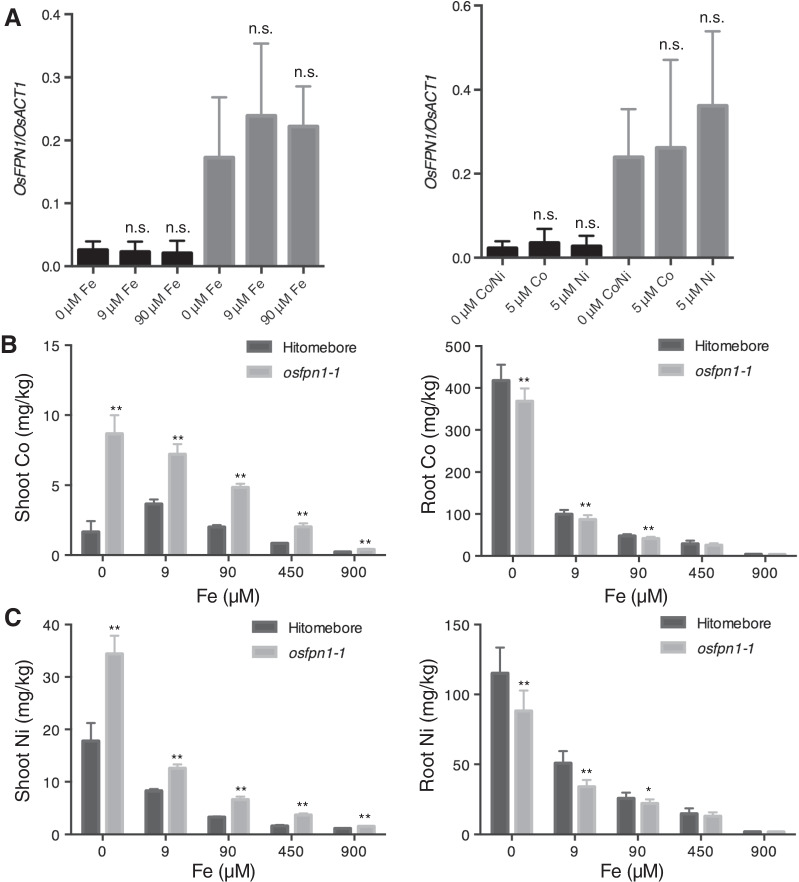
Effect of Fe on Co and Ni accumulation. **A** *OsFPN1* expression levels in the shoots (black) or roots (gray) of 10-d-old seedlings under 0, 9, and 90-µM Fe-citrate conditions or 0 and 5-µM Co or Ni conditions (n = 4). *OsACT1* is *OsActin1.* Dunnett’s test (compared to 0 µM Fe or 0 µM Co/Ni in the shoot or root). n.s., *p* ≥ *0.05.* Tukey’s multiple-comparison tests were conducted among the same tissue, *p* < 0.05. Co (**B**) and Ni (**C**) concentrations in the shoots (left panel) and roots (right panel) of 21-d-old seedlings. The x-axis represents the Fe concentration (µM). Plants were grown in a hydroponic culture. Data represent the mean ± SD (n = 8–10). Student’s *t*-test (compared to Hitomebore). *p < 0.05; **p < 0.01

In *Arabidopsis*, two genes are involved in Co and Ni transport: *AtFPN1* and *AtFPN2* (Morrissey et al. 2009). The mRNA expression of *FPN2* is upregulated by low levels of Fe (Morrissey et al. 2009), whereas that of *AtFPN1* is not (Morrissey et al. 2009). *AtFPN2* can sequester Co and Ni into vacuoles in roots, while *AtFPN1* can export Co to the shoot. To confirm whether *OsFPN1* mRNA is regulated by Fe, Co, and Ni, rice was grown under 0, 9, and 90 μM of Fe, or 0 and 50 μM of Co or Ni in Kimura B solution and the *OsFPN1* mRNA levels were quantified. Unlike *AtFPN2*, the mRNA expression of *OsFPN1* was not altered by Fe, Co, or Ni (Fig. 7A).

In *Arabidopsis*, the difference in the Co concentration in the shoots of the wild type and *fpn2* increased under low-Fe conditions (Morrissey et al. 2009). To investigate whether Fe affected Co and Ni accumulation between the wild type and *osfpn1-1*, we analyzed the accumulation of Co and Ni in their shoots and roots under various Fe conditions. There was no large difference in growth under the tested conditions (Additional file 1: Fig. S5). Co was higher in *osfpn1-1* under most of the Fe conditions than in the wild type (Fig. 7B). However, the opposite scenario was observed in the roots. The difference in Co accumulation in the shoots between the wild type and *osfpn1-1*/*1187_n* was larger under 0 μM Fe conditions than under normal Fe (90 µM) conditions, as well as the difference in the Co accumulation in roots (Fig. 7B). A similar pattern was observed for Ni (Fig. 7C). These results indicated that OsFPN1 is involved in the root-to-shoot translocation of Co and Ni, particularly under low-Fe conditions.

## Discussion

Through ionome screening, we isolated *osfpn1-1* and identified *OsFPN1* as a gene responsible for Co and Ni accumulation, as well as for tolerance to high Co or Ni conditions. It has been reported that plant FPN has a wide substrate specificity. For instance, Arabidopsis *FPN2* and poplar *PgIREG1* can transport Co or Ni (Morrissey et al. 2009; Merlot et al. 2014; Yokosho et al. 2016). In buckwheat, *FeIREG1* is involved in Al detoxification (Yokosho et al. 2016). In this study, heterologous expression of *GFP-OsFPN1* in yeast increased the sensitivity to high concentrations of Co (Fig. 6B). Furthermore, the yeast experiment suggested the Co and Ni transport activities of *OsFPN1* (Fig. 6C). These results suggest that OsFPN1 can transport Co and Ni in rice.

In the yeast experiments, the yeast carrying *OsFPN1* exhibited increased sensitivity to high Co and accumulated less Co and Ni than the empty vector. In a previous report on Arabidopsis FPN, it was suggested that FPN proteins could be transported from the cytosol to the outside of the cell or into the vacuole (Morrissey et al. 2009). *OsFPN1* in yeast is localized to a dot-like structure, as well as the plasma membrane (Fig. 6A). Considering the direction of transport and plasma membrane localization, *OsFPN1* transports Co^2+^ and Ni^2+^ to the outside of the cell, leading to low Co^2+^ and Ni^2+^ contents (Fig. 6C). In contrast, the yeast exhibited high Co sensitivity (Fig. 6B). This is contradictory to the result of the yeast Co and Ni concentrations because, if Co is exported to the outside of the cell, the yeast would exhibit tolerance, similar to the *COT1*-expressing yeast. The reason for this is currently unknown, but may be due to the high accumulation of Co in a dot-like organelle, such as the Golgi. Inside the organelle, the Co^2+^ and Ni^2+^ contents would be sufficiently high to inhibit the reaction, leading to growth inhibition.

*OsFPN1* was localized to the Golgi when transiently expressed in protoplasts prepared from rice (Fig. 5). In *Arabidopsis*, *FPN1* and *FPN2* are localized to the plasma membrane and vacuole, respectively (Schaaf et al. 2006; Morrissey et al. 2009). In our experiments, both C-terminal and N-terminal fusion proteins were localized to the Golgi in rice, and *GFP-OsFPN1* exhibited Co and Ni transport activity in yeast (Figs. 5, 6). These results indicate that *GFP-OsFPN1* is functional and that the fusion protein reflects the function and localization of endogenous *OsFPN1*. In plants, there are currently no reports indicating that the Golgi are a storage site of Co. It has been reported that Co highly accumulates in the perinuclear fraction of human keratinocytes, and can accumulate in the endoplasmic reticulum or Golgi (Ortega et al. 2009). Therefore, the Golgi apparatus might be a storage site for Co in rice.

## Conclusion

Our study shows that *OsFPN1* mediates Co and Ni transport, and is involved in the detoxification of Co and Ni. From the viewpoint of application, a high Co content in grains and leaves would be useful for the biofortification of grasses as it would resolve Co deficiencies in sheep and cattle.

## Materials and Methods

### Plant Materials and Growth Condition

The ionome mutant, hereafter termed *1187_n*, was isolated from EMS-mutagenized *Oryza sativa* cv. Hitomebore in a previous study (Tanaka et al. 2016). For hydroponic cultivation, surface-sterilized seeds of rice were sown in a germination solution [0.19 mM CaCl_2_ ·2H_2_O, 2 mM MES (pH = 5.8)] for one week, transferred to Kimura B solution (0.35 mM (NH_4_)_2_SO_4_, 0.47 mM MgSO_4_•7H_2_O, 0.54 mM KNO_3_, 0.18 mM Ca(NO_3_)_2_•4H_2_O, 0.17 mM Na_2_HPO_4_•12H_2_O, 90 μM Fe-citrate, 0.19 mM CaCl_2_, 4.6 μM MnSO_4_•5H_2_O, 0.18 μM H_3_BO_3_, 0.10 μM Na_2_MoO_4_•2H_2_O, 0.15 μM ZnSO_4_•7H_2_O, and 0.16 μM CuSO_4_•5H_2_O) supplemented with 0.1 μM of Li, Rb, Cs, Sr, Cd, As, Se, Co, Ni, and Ge, and grown for an additional three weeks in a growth chamber at 28 °C under 16 h light/8 h dark conditions. For the Co, Ni, and Fe treatment, NiCl_2_, CoCl_2_, and Fe-citrate were supplied, respectively. In all hydroponic experiments, the solution was renewed every 7 d. For the seeds grown in soil, the surface-sterilized seeds were germinated in tap water for one week and then transplanted into commercial soil (Honensu soil, Kumiai Kagaku) and grown for an additional three weeks in a growth chamber at 28 °C under 16 h light/8 h dark conditions.

### Element Determination

The plants were separated into shoots and roots and washed with ultrapure water. After drying at 70 °C for 3 d, the dry weights of the shoots and roots were measured and digested with HNO_3_. After dissolving the digested samples in 0.08 M HNO_3_ containing 2 ppb of In (internal control), the element concentrations were determined by Inductively coupled plasma-mass spectrometry (ICP-MS) (Agilent, Agilent 7800).

### Genotyping of F_2_ Crosses Between*** 1187_n*** and Hitomebore

*1187_n* was backcrossed with wild-type Hitomebore, and a self-fertilized F_2_ population was used to confirm the correlation between the phenotype (Co and Ni) and genotype. Genotyping was conducted using dCAPS markers and primers Nos. 1 and 2 (Additional file 1: Table S1).

### Plasmid Constructs and Transformation

For the subcellular localization of OsFPN1, coding sequences (CDS) were amplified with primers Nos. 7 and 8 for GFP-OsFPN1, or Nos. 7 and 9 for OsFPN1-GFP (Additional file 1: Table S1). The amplified fragment was ligated into the EcoRI and XhoI sites of the pENTR2B-dual entry vector (Life Technologies) using a DNA ligation kit (Ligation High ver.2, TOYOBO). CDS was cloned into pMDC45 or pMDC85 vectors (Curtis and Grossniklaus 2003) using LR clonase (Life Technologies). For OsCTL1-red fluorescent protein (RFP), a Golgi marker, CDS was amplified using primers Nos. 10 and 11 (Additional file 1: Table S1). The CDS was ligated into the XbaI and SalI sites of the pENTR2B-dual entry vector using a DNA ligation kit (Ligation High ver.2, TOYOBO). CDS was cloned into the destination vector pGWB554 (Nakagawa et al. 2009), which contains RFP, using LR clonase (Life Technologies).

To generate CRISPR/Cas9 mutant lines of *OsFPN1,* 20-nt (primers Nos. 14 and 15; Additional file 1: Table S1) targeting the gene was annealed and introduced into the pU6gRNA vector (Mikami et al. 2015). pU6gRNA was digested with AscI and PacI, and the digested fragment was inserted into the AscI and PacI sites of pZH_OsU6gRNA_MMCas9 (Mikami et al. 2015). The construct was transformed into *Agrobacterium tumefaciens* EHA105 and used for rice transformation (Ozawa 2012). *Oryza sativa* cv. Nipponbare was used for transformation in all experiments, and mutations in *OsFPN1* CRISPR/Cas9 mutants were identified using primers Nos. 16 and 17 by direct sequencing (Additional file 1: Table S1).

To generate an expression vector expressing GFP-COT1p in the yeast experiments, the GFP fragment was amplified with primers Nos. 18 and 19, and the CDS of *COT1* was amplified with primers Nos. 12 and 13 (Additional file 1: Table S1). GFP and COT1 fragments were directly cloned into the XhoI and SalI sites of pKT10-myc (Tanaka et al. 1990) using an In-Fusion® HD Cloning Kit (TOYOBO). GFP-OsFPN1 was amplified with primers Nos. 20 and 21 (Additional file 1: Table S1) from pMDC45 carrying GFP-OsFPN1, and the fragment was cloned into the EcoRI and SalI sites of pKT10-myc using a DNA ligation Kit (Ligation High ver.2, TOYOBO).

### Confocal Laser Scanning Microscopy Observation

Plasmids of GFP-OsFPN1 or OsFPN1-GFP were co-transformed with OsCTL1-RFP into 10-d-old rice protoplasts prepared from leaf sheaths grown in 0.5 × MS medium following the protocol described by Zhang (Zhang et al. 2011). After incubation in W5 solution (154 mM NaCl, 125 mM CaCl_2_, 5 mM KCl, and 2 mM MES at pH 5.7) at 25 °C for 16–20 h (Wu et al. 2012), the fluorescence of GFP and RFP was observed by confocal microscopy (FV1000, Olympus). The excitation and emission wavelengths were set at 473 nm and 500–525 nm for GFP and 559 and 600–660 nm for RFP, respectively.

### Yeast Growth Assay and Element Uptake Experiments

The plasmids were introduced into Co- and Ni-sensitive mutants, *cot1* (*MATa, ura3Δ0, leu2Δ0, his3Δ1, met15Δ0, and YOR316c::kanMX4*) (Conklin et al. 1992). After transformation, two independent colonies were isolated and used in the experiments. The yeast was cultivated overnight in SD uracil-free medium, and the OD_600_ was adjusted to 1.0. After a series of dilutions, the yeast was spotted onto SD uracil-free media supplemented with 1-mM Co or without Co and incubated at 30 °C.

To determine the elements in yeast, the yeast was incubated overnight and adjusted to the same optical density (OD_600_ = 0.3), and then incubated in SD uracil-free liquid medium supplemented with 50 μM of Co or 50 μM of Ni for 1 h. After incubation, the yeast cells were washed three times with cold ultrapure water and dried overnight at 70 °C. The dried cells were then digested with HNO_3_ at 70 °C for 1 h. After dilution with 0.08-M HNO_3_ containing 2 ppb of In, the samples then underwent ICP-MS analysis.

### RNA Extraction and Expression Analysis

The total RNA was extracted from 10-d-old seedlings using an RNeasy Plant Mini Kit (Qiagen) following the manufacturer’s instructions. Approximately 500 ng of the total RNA was reverse-transcribed to cDNA using a PrimeScript RT reagent kit (Takara), which was then diluted 10 times and used for quantitative (q) PCR using TB Green® Premix Ex TaqTM II (Tli RNaseH Plus, Takara). *OsActin1* (Zhang et al. 2004) was used as an internal control for qPCR. Nos. 3 and 4, and Nos. 5 and 6 primers were used for the qPCR of *OsFPN1* and *OsActin1*, respectively (Additional file 1: Table S1).

### Statistical Analysis

The sample size is described in the figure legend and statistical analysis was performed with GraphPad Prism software (https://www.graphpad.com/scientific-software/prism/).

## Supplementary Information

**Additional file 1: Figure S1**. Ionome pattern of *1187_n*. **Figure S2**. Phylogenetic tree of FPN. **Figure S3**. Structure of *OsFPN1* protein. **Figure S4**. Spatio-temporal gene expression of various tissues/organs throughout entire growth in the field. **Figure S5**. Growth phenotype of Hitomebore and *osfpn1-1* under various Fe conditions. **Table S1**. Primers used in this study.

## Data Availability

All data generated or analysed during this study are included in this published article and its Additional files.

## Abbreviations

CDS: Coding sequences
FPN: Ferroportin
GFP: Green fluorescent protein
ICP-MS: Inductively coupled plasma-mass spectrometry
IREG1: Iron-regulated gene1
RFP: Red fluorescent protein
CTL1: Chitinase-like1
EMS: Ethyl methanesulfonate
