# Supplementary material for: Golgi-Localized OsFPN1 is Involved in Co and Ni Transport and Their Detoxification in Rice
Source: Rice (N Y). 2022 Jul 11;15:36. doi: 10.1186/s12284-022-00583-3 (PMC9273799; doi:10.1186/s12284-022-00583-3)
Supplement: Supplementary file 1 — Additional file 1: Figure S1. Ionome pattern of 1187_n. Figure S2. Phylogenetic tree of FPN. Figure S3. Structure of OsFPN1 protein. Figure S4. Spatio-temporal gene expression of various tissues/organs throughout entire growth in the field. Figure S5. Growth phenotype of Hitomebore and osfpn1-1 under various Fe conditions. Table S1. Primers used in this study. [file 12284_2022_583_MOESM1_ESM.pdf]

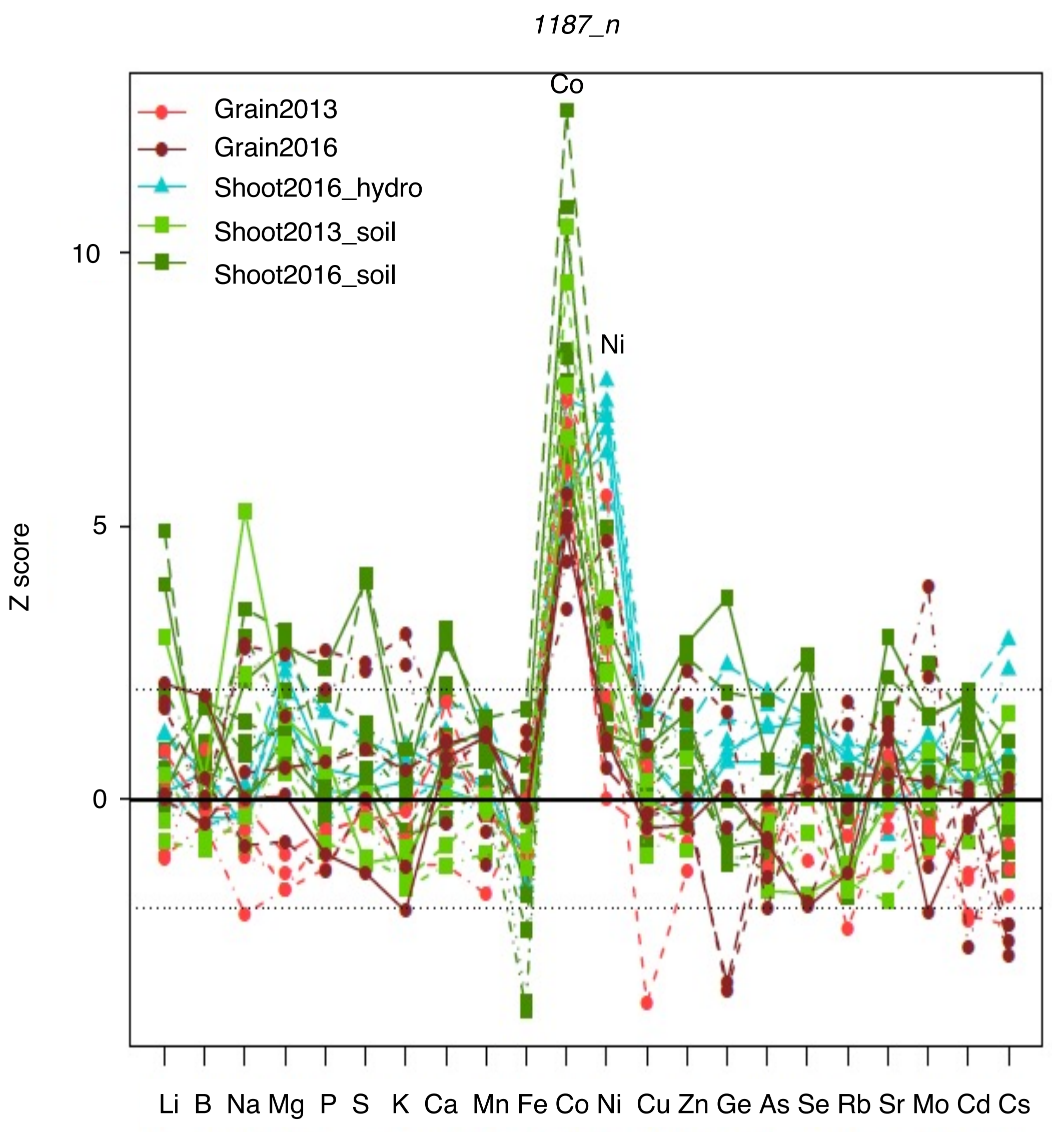

Figure S1. Ionome pattern of *1187_n*
Ionome profiles of the shoots and grains of *1187_n* described as Z scores compared to those of Hitomebore in 2013 and 2016. Red, dark-red, aqua, green, and dark-green represent the Co and Ni concentrations in the grains grown in paddy fields in 2013, grains grown in paddy fields in 2016, shoots frown in Kimura B solution supplied with 10 trace elements in 2016, shoots grown in soil in 2013, and shoots grown in soil in 2016 (n = 3–5).

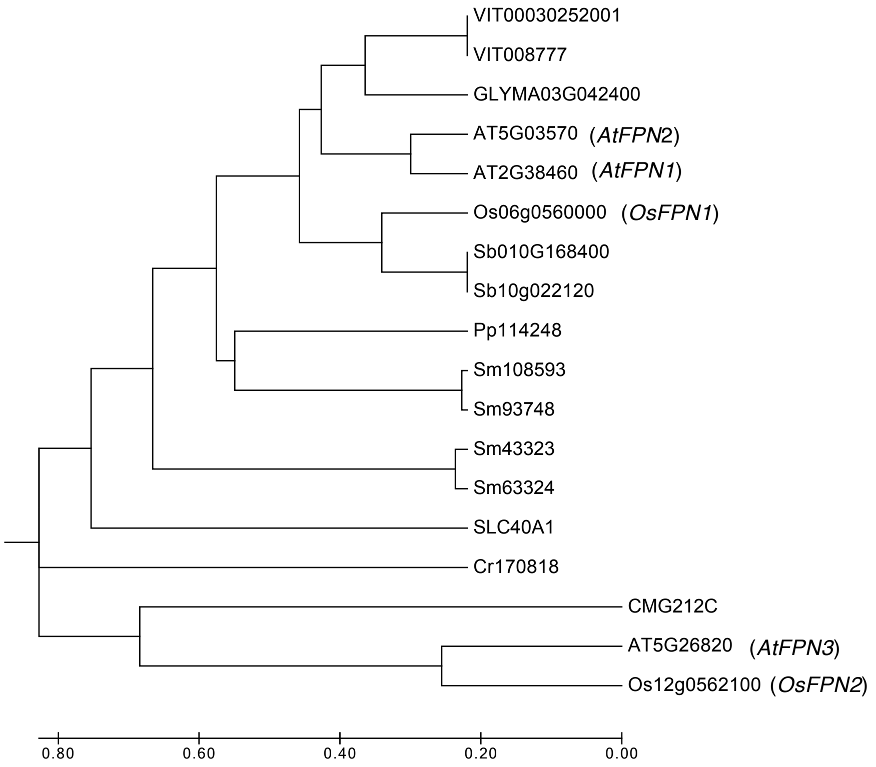

Figure S2. Phylogenetic tree of FPN.

Phylogenetic tree based on amino acids VIT, *Vitis vinifera*; Sb, *Sorghum bicolor*; GLYMA, *Glycine max*; Pp, *Pyscomitrella patens*; AT, *Arabidopsis thaliana*; Sm, *Selaginella moellendorffii*; SLC, *Homo sapiens* (humans); Os, *Oryza sativa*; Cr, *Chlamydomonas reinhardtii*; CM, *Cyanidioschyzon merolae*.

A

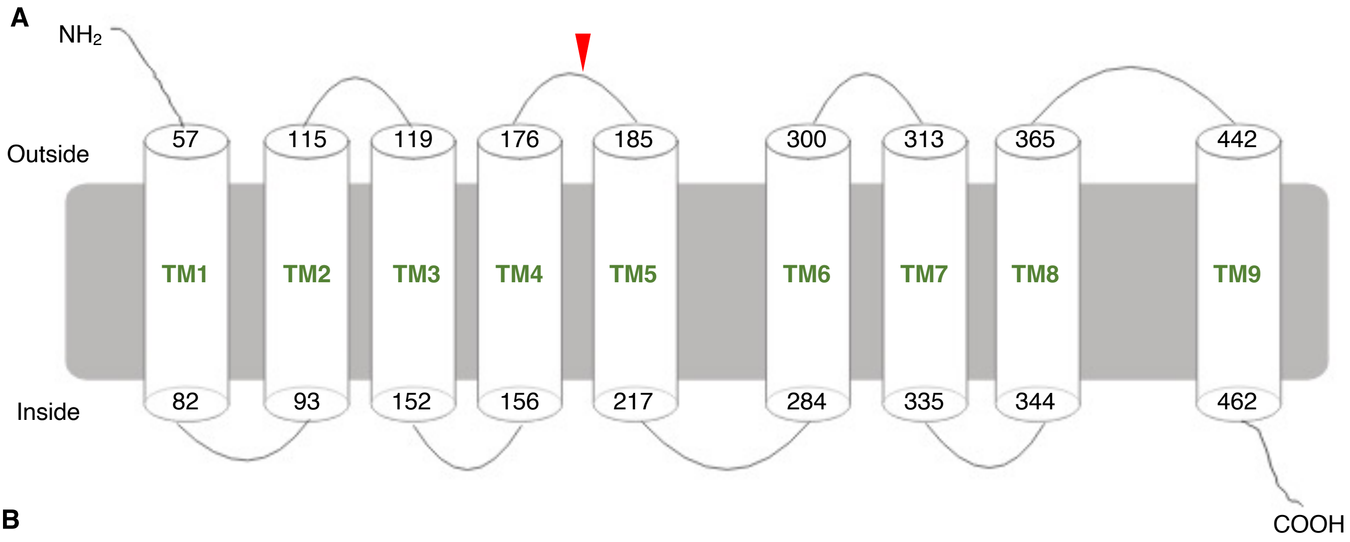

B

```
                          TM4                              TM5
OsFPN1     165  PAAVLTGINSVIRRIDLSCKLLAPVLSGFFISFVSMEASAAALAAWNLAAVWVQYWLFVS
AtFPN2     171  SPAVLTRMNSVIRGIDLSSKLLSPVITGLIISFVSLRASAITFAAWATITVWIEYWLFIS
AtFPN1     178  PPAVLTKMNSVIRGIDLSSKLLSPVITGLIISFVSLKASAITFAAWATITAWVEYWLFIS
OsFPN2     277  RPVALAQANAVLNRLDLVCETVGASVFGLLLSKYHP-VTCLKIACGLMICSFPVLVVLGQ
AtFPN3     287  RPIALAQANAVLHRIDLLCEIAGTMLFGILLSKYDP-VTCLKFAATLMVGSLPTMTALIW
HsFPN1     301  SYYNQPVFLAGMGLAFLYMTVLGFDCITTGYAYTQGLSGSILSILMGASAITGIMGTVAF
consensus  301   pavlt in virridLscklllgpvlsgliisfvsm asai faawaliavwv ywlfis
```

Figure S3. Structure of the *OsFPN1* protein.
(A) Model of *OsFPN1*. Putative transmembrane domains of *OsFPN1* were predicted by https://embnet.vitalit.ch/software/TMPRED_form.html. TM, transmembrane. (B) Partial alignment of FPNs. https://embnet.vital-it.ch/software/BOX_form.html. Amino acids colored in green are TM4 and TM5. Red triangle is a mutation site in *osfpn1-1*.

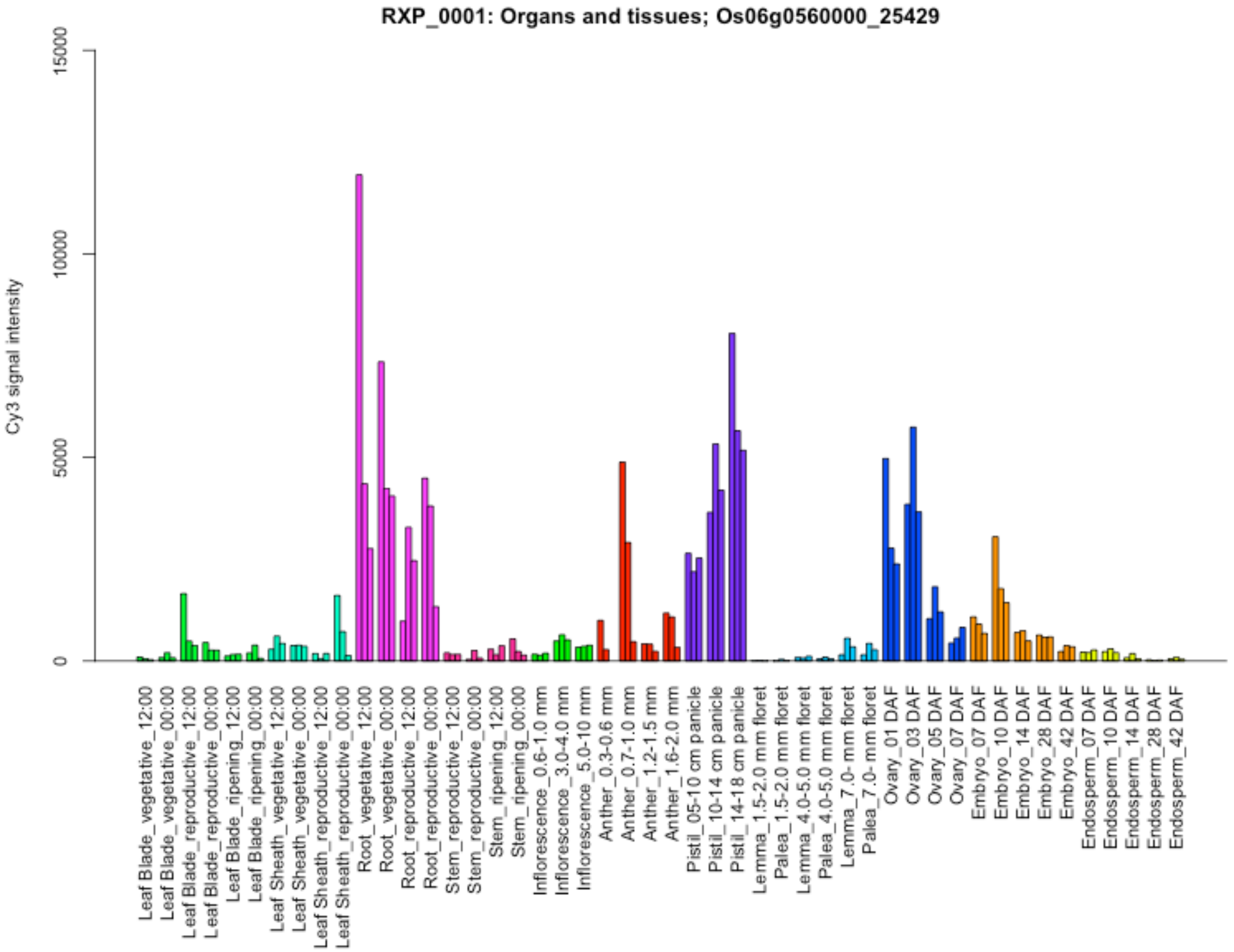

Figure S4. Spatio-temporal gene expression of various tissues/organs throughout entire growth in the field.

The data was obtained from RiceXPro database (https://ricexpro.dna.affrc.go.jp/) (Sato et al. 2011)

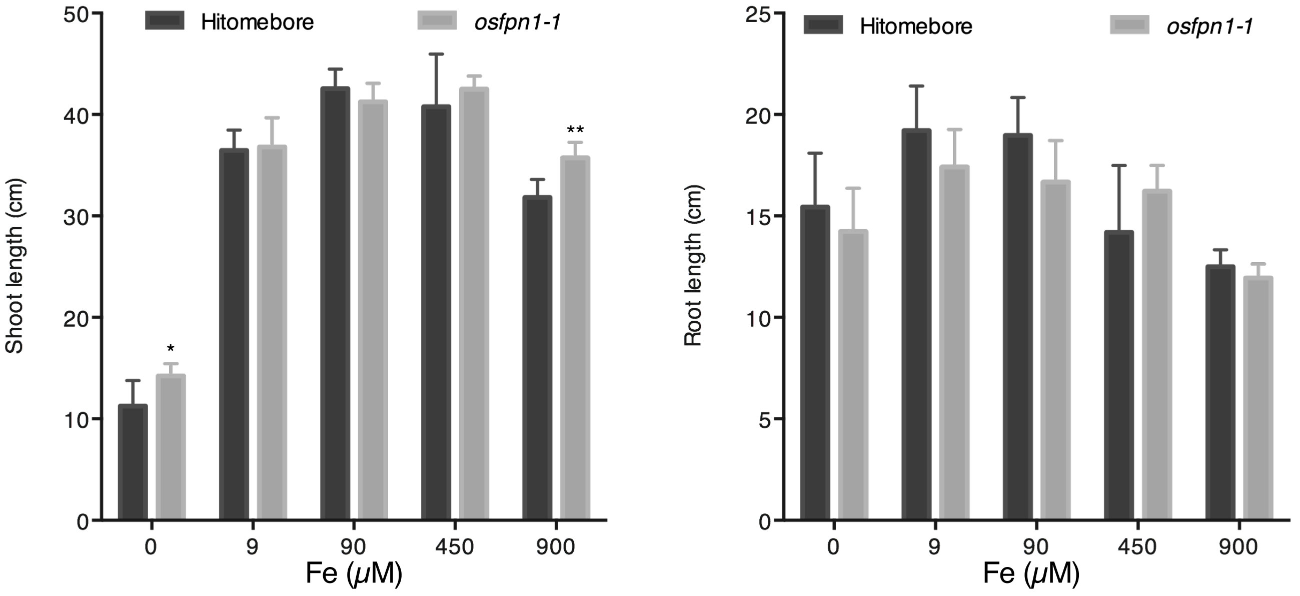

Figure S5. Growth phenotype of Hitomebore and *osfpn1-1* under various Fe conditions
Shoot and root lengths under under 0, 9, and 90 μM Fe-citrate conditions. Plants were grown in a hydroponic culture for three weeks. Data represent the mean ± SD (n = 5–10). Student's t-test, *, $p < 0.05$; **, $p < 0.01$.

Table S1. Primers used in this study.

Primer name	sequence	Note
No.1	GAATTTGCTGCGTGCAGGGTGGTGG	dCAPS marker of Os06g0560000
No.2	ACAGCACCGGCGCCAGCAGCTTGCAGATC	
No.3	CCTCTGATCCGTACATCTTCTTG	qPCR of Os06g0560000
No.4	CCATCACTCGCCATACTTACTC	
No.5	GTATCCATGAGACTACATACAACT	qPCR of OsACT1
No.6	TACTCAGCCTTGGCAATCCACA	
No.7	CGCCGGAATTCATGGCGAGTGATGGCCACCTCGC	amplify CDS of Os06g0560000 (with stop codon)
No.8	CGCCGCTCGAGTCAAGAAGCTTTGATCCAGTTCATC	
No.7	CGCCGGAATTCATGGCGAGTGATGGCCACCTCGC	amplify CDS of Os06g0560000 (without stop codon)
No.9	CGCCGCTCGAGGCAGAAGCTTTGATCCAGTTCATCTTG	
No.10	TTCAGTCGACATGAAGAGGAAGACTCGGAAC	amplify CDS of OsCTL1 (with stop codon)
No.11	CGC CCGTCTAGAGCGCTTTGTTGCTGCTGATCGTC	
No.12	AAAGGCGCGCCAAGCTATCACATGAAACTCGGAAGCAAACAGG	amplify COT1 (with stop codon)
No.13	ACGTGTTCAACCAAGTCGATTAATGATCCTCTAAGCAATCAGC	
No.14	GTTGTACATGATCCGAATCTGGCC	gRNA of Os06g0560000 for CRISPR/ Cas 9
No.15	AAACGGCCAGATTCGGATCATGTA	
No.16	ATCGGAGTGATCGATCAGTC	identification the mutation of CRISPR line *osfpn1-2* and *osfpn1-3*
No.17	CATGGACACGAAGCTGATGAAG	
No.18	TCCGAAGAAGATCTGGAATTATGAGTAAAGGAGAAGAACTTTTC	amplify GFP
No.19	GTGATAGCTTGGCGCGCCTTTGTATAGTTCATCCATGCCATGTG	
No.20	CGCCCGCTCGAGATGAGTAAAGGAGAAGAACTTTTC	amplify of GFP-OsFPN1
No.21	CGCCCGTCTAGATTA ATG ATC CTCTAAGCAATCAGC	
